# From All Quarters

**Published:** 1910-05-14

**Authors:** 


### FROM ALL QUARTERS.

Owing to the lamented death of H.M. King Edward VII. the performance at the Royal Court Theatre on Monday next has been postponed.

---

The Royal Hospital for Incurables, Putney Heath, has received from Mrs. Ellen Whitwham a special donation of five guineas "In memory of King Edward," who was a patron of the hospital for thirty years.

---

The Secretary of the Royal Society of Medicine announces that in consequence of the death of his late Majesty the King, Patron of the Society, all ordinary meetings of the Society, and of the sections, are postponed until after the King's funeral.

---

Owing to the death of the King the special meeting of the Royal National Orthopædic Hospital, to have been held at the Mansion House on Thursday last and presided over by the Lord Mayor, will not be held. There is a debt of £17,000 on the hospital, which his late Majesty opened last July, and of which he was patron. No one will desire the interests of the charity or the welfare of the crippled and deformed children to suffer, and any donations will be gratefully received.

---

In consequence of the lamented death of his Majesty the King, the patron of the Royal Institution of Great Britain, and out of respect to his memory, the President has decided that the lectures and evening meetings here shall be discontinued until further notice. A general monthly meeting of the Institution was held on Monday last, the Duke of Northumberland, President, in the chair. The following gentlemen were nominated Vice-Presidents for the ensuing year :—Sir Thomas Barlow, Sir H. Burton Buckley, Sir William Huggins, O.M., Mr. A. B. Kempe, Sir Francis Laking, Mr. George Matthey, Sir James Crichton-Browne (treasurer), and Sir William Crookes (honorary secretary). The meeting adjourned to May 23, when a resolution of condolence on the occasion of the lamented death of King Edward, patron of the Royal Institution, will be passed.

In consequence of the death of King Edward VII., Patron of the League and Sovereign of the Order of Mercy, there will be no meetings held next week in connection with the League of Mercy.

---

It has been decided that the arrangements already made for a large number of Territorial troops to begin their fortnight's camp training next Saturday shall not be stopped by the national mourning.

---

A proclamation published in the London *Gazette* on Tuesday last directs that Friday, the 20th day of May, shall be observed as a day of General Mourning throughout the United Kingdom and every part thereof.

---

Owing to the national calamity and sad loss of the Royal patron of the Italian Hospital, the ball arranged in aid of its funds for the 24th inst. has been cancelled. Holders of tickets desiring their money refunded can have it on application.

---

Mr. W. F. Cochrane, secretary of the German Hospital, Dalston, of which institution his late Majesty King Edward VII. was a patron and generous friend, announces that in consequence of the much lamented death of his Majesty the annual (65th anniversary) festival dinner of the hospital, fixed for Thursday, the 19th inst., has been abandoned.

---

The following hospital and other arrangements, among others, have been cancelled or postponed in view of the King's death : Annual view at St. Bartholomew's Hospital (cancelled) ; Gordon Hospital annual meeting (postponed to May 26) ; General meeting Royal College of Physicians (postponed to May 26) ; University of London graduation day (postponed) ; Annual dinner King's College (postponed) ; Annual dinner Royal Sanitary Institute (postponed).

The King's Fund has received a special donation to capital of £50 from Mr. W. D. Henriques as a tribute to the memory of King Edward VII.

---

"That in consequence of the death of his late Majesty King Edward VII. the Festival of Empire be not opened this year, in the hope that it may take place next year under happier auspices."

---

Owing to the lamented death of his late Majesty, the special *matinée* which was to take place at His Majesty's Theatre on May 23, on behalf of the Royal Waterloo Hospital, has been postponed.

---

The following note appears in the *Times* of last Tuesday: Though underwriters at Lloyd's are affected to a certain extent by insurances maturing on the death of the late King, it is understood that the great bulk of this business has been placed with the life insurance offices. It is customary every year for the companies to receive a large number of proposals connected with the life of the Sovereign and relating to all descriptions of business, from the falling-in of leases to losses arising from stocks of Prayer-books becoming out of date. The rates quoted are usually based on the ordinary mortality tables, and are dependent, of course, on the latest reports regarding the health of the Royal life assured. The insurances are either annual contracts or for temporary periods covering some important event, and there is no difficulty in effecting them provided the assured can prove his interest.

---

Mr. J. C. Bodley, writing in Monday's *Times*, points out that the late King, when Prince of Wales, joined the Royal Commission on the Housing of the Working Classes in 1884. Mr. Bodley gives some interesting reminiscences in the course of his letter.

"His Royal Highness attended and sat out fourteen of its long sittings. Moreover, his attendances would have nearly reached the *maximum* but for the death of the lamented Duke of Albany, which kept his most affectionate brother away from his public duties for two months. In 1885 the evidence for England came to an end, after which no record of attendance was kept. But I have reason to remember how regularly his late Majesty came to the bi-weekly meetings, as it then became my duty to read aloud to the Commissioners my draft report, portions of which they criticised at each sitting. The late King's criticisms were always pertinent, as were his questions to witnesses, whom he examined with remarkable grasp of the subject. He used to interrogate not only witnesses of public celebrity, such as the venerable Lord Shaftesbury or Mr. Chamberlain (when he gave his famous evidence on "unearned increment"), but also modest workers among the poor, such as was then a Buckinghamshire vicar who is now Bishop of Truro. On the same day as Dr. Stubbs, the late Mr. Beck, the agent at Sandringham, was called, and testified to what had been done for the labourers on that estate. Not content with letting us hear his agent, his Royal Highness, on several occasions, invited some of us to Sandringham to see for ourselves how the housing problem had been anticipated on the Royal domain. Lord Carrington, who was a member of the Commission, will recollect his late Majesty's adventure in the East End, when, resolved to see for himself how the poor of London lived, he disguised himself in a strange ulster and a slouch hat so well as completely to deceive a familiar vestryman who acted unknowingly as guide to the Heir to the Throne."

The annual meeting of the East End Mothers' Lying-in Home has been postponed until after the late King's funeral.

---

In consequence of the death of His Majesty King Edward, patron of the Society, no meeting of the Royal Society will be held this month.

---

"Printers' Pie: 1910."—As a mark of respect for our late Gracious King, Mr. W. H. Spottiswoode has decided to postpone the publication of "Printers' Pie" until June 7 next.

---

In consequence of his late Majesty the King's death, nearly all hospital functions, festival dinners, and meetings are postponed until after the funeral. In many cases certain functions have been indefinitely postponed.

---

On all the hospitals, both metropolitan and provincial, flags were at half-mast early on Saturday morning. It was interesting to notice that contrary to the custom in some quarters, the hospital Union Jacks were all hoisted correctly, and not upside down.

---

King Edward's annual subscription of ten guineas to the Royal Westminster Ophthalmic Hospital was posted the day before his death, and the bank has informed the hospital that the cheque will be duly honoured. This was probably his Majesty's last gift to a public institution.

---

In consequence of the lamented death of his Majesty the King, the festival dinner of the City of London Hospital for Diseases of the Chest, Victoria Park, E., of which his late Majesty was a patron and generous supporter, fixed for the 12th inst., has been postponed.

---

His Serene Highness Prince Francis of Teck ordered an Extraordinary Court of Governors of the Middlesex Hospital to be held on Friday, the 13th inst., at 3 p.m., for the purpose of voting a humble address of condolence to his Majesty the King on the lamented occasion of the death of his late Majesty King Edward VII., patron of the hospital.

---

Various suggestions have been made as to the manner in which the citizens of London should show their sympathy with the Royal Family. It is generally acknowledged that on the day of the funeral all public and private places of business should be closed. Now, however, it has been proposed that restaurants and tea shops should be open on that day and that the proceeds should be handed over to the King's Fund.

---

Alderman Thompson, of Richmond, presided at the informal opening of the second Municipal and Health Exhibition at the Royal Agricultural Hall, Islington, last Saturday. He said that King Edward was not only the greatest King, but the greatest sanitarian this country had ever known. The exhibition had been organised to promote municipal work by bringing under one roof all the latest appliances and inventions for sanitation, roadmaking, and building, with special regard to cottages for the working classes, and to give the ratepayers an opportunity of seeing how their money could be rightly expended. He maintained that sanitary science worked in the end for the reduction of the rates. The exhibition will remain open until to-day, Saturday.

